# Efficacy of Crest Herbal Toothpaste in “Clearing Internal Heat”: A Randomized, Double-Blind Clinical Study

**DOI:** 10.1155/2013/807801

**Published:** 2013-10-20

**Authors:** Jia-Xu Chen, Yue-Yun Liu, Shao-Xian Wang, Xiao-Hong Li

**Affiliations:** School of Preclinical Medicine, Beijing University of Chinese Medicine, No. 11, Beisanhuan Donglu, Chaoyang District, P.O. Box 83, Beijing 100029, China

## Abstract

*Objective*. Evaluation of the efficacy of Crest Herbal Crystal Toothpaste in “clearing internal heat.” *Methods*. This was a randomized, double-blind, controlled parallel design clinical test of a product that was already on the market. 72 subjects were randomly assigned to control group (group A with Colgate Herbal Salty Toothpaste) or treatment group (group B with Crest Herbal Crystal Toothpaste) with ratio of 1 : 2. Subjects were instructed to brush with 1g toothpaste for 2 minutes each time, 2 times per day in a 4-aweek test period; measurement with the rating scale on the efficacy of “clearing internal heat” for the herbal toothpaste was done at baseline, 2 weeks, and 4 weeks of toothpaste usage. *Results*. The rating scale on efficacy of “clearing internal heat” for the herbal toothpaste reveals that the primitive points of 72-case intention-to-treat (ITT) analysis and 67-case per-protocol (PP) analysis for subjects in group A and subjects in group B were found to be reduced progressively with statistical significance (*P* < 0.05). The overall effective rates for group A and group B were, respectively, 62.50%, 56.25% (ITT) and 62.50%, 60.64% (PP). The statistical results indicated that the symptoms of fire-heat for both groups of subjects have been improved after application of toothpaste. *Conclusion*. The efficacy of Crest Herbal Crystal Toothpaste in “clearing internal heat” was confirmed by the trial as compared to Colgate Herbal Salty Toothpaste. And its efficacy was objectively evaluated by the rating scale on efficacy of “clearing internal heat.”

## 1. Introduction

With the quickened pace of life and increased pressure of work for nowadays people, oral diseases such as toothache, sore, or bleeding gums occur very commonly. Most of these symptoms were considered “excessive internal heat” by traditional Chinese medicine (TCM). Meanwhile, as one of the daily necessities of oral cleaning agents, many types of herbal toothpaste claim to have the efficacy of “clearing internal heat.” When consumers select toothpastes, their decisions were not only based on cleaning factors, but also on considerations for dental care and prevention of oral diseases. Because of cultural and geographical factors, herbal toothpastes with efficacy of “clearing internal heat” were estimated to have enormous market opportunities in China. Currently, there were a few reports [1, 2] on the efficacy of “clearing internal heat” by TCM for oral diseases, and effectiveness of herbal toothpastes has been found. 

Traditional Chinese medical science paid extra attention to dental hygiene and prevention and treatment of diseases at early time. Totally eight categories were documented in the sections that involve oral diseases in Prescriptions for Universal Relief in Ming Dynasty (early at 15 centuries BC) [3], and ancient TCM doctors had already accumulated very abundant experiences in the prevention and treatment of oral diseases. 

Crest Herbal Crystal Toothpaste, containing the essence scientifically extracted from the natural herbs of honeysuckle and mint with function of anti-inflammation and sterilization as well as heat-clearing and detoxication, is a kind of toothpaste of TCM exclusively manufactured by P&G Technology Co., Ltd, which has been identified to effectively remove bacteria in the mouth and freshen breath to prevent gum disease, therefore, keeping overall health for teeth and gums.

The purpose of this clinical trial was to scientifically evaluate the efficacy of Crest Herbal Crystal Toothpaste, using our reported rating scale (the 3rd edition) [4] with good reliability and validity on efficacy of “clearing internal heat” for the herbal toothpaste developed in prophase. The selected positive control toothpaste was Colgate Herbal Salty Toothpaste with effect of “clearing internal heat,” containing herbal essence, of which the main ingredients were honeysuckle and mint for tingly fresh breath and all day protection, soothing oral ailments like toothaches, mouth ulcers, and inflamed gums. The control product has been in the China market for a long time with the following claim: reducing internal heat. We therefore chose them as a comparison to the Crest brand.

## 2. Data and Methods

### 2.1. Demographic Data

All subjects were recruited by the Research Institute of Crest Oral Care, Beijing P&G Technology Co., Ltd., and the trial period was from August 26, 2009 to September 23, 2009. 72 subjects were randomly divided by ratio of 1 : 2 into control group with 24 subjects (group A) and test group with 48 subjects (group B). As it was the first time to evaluate the “clearing internal heat” efficacy of Crest herbal product, more data were needed to get a clear understanding relative to the control product, so 1 : 2 ratio recruiting was used in this study.

72 subjects were typical permanent residents in Beijing, among whom were 16 of males and 56 of females, aged between 19 and 55, with average age of 28, and randomly divided into two groups with the ratio of 1 : 2, and toothpaste A or toothpaste B was provided, respectively, where 24 subjects were arranged in group A and 48 subjects in group B. After unblinding, it was found that Colgate Herbal Salty Toothpaste was provided for group A, while Crest Herbal Crystal Toothpaste was for group B. The period of using the toothpaste was 4 weeks, collecting data of oral cavity and related symptoms of oral conditions for subjects after the end of the second week and the fourth week. 

### 2.2. Diagnostic Criteria and Inclusion and Exclusion Criteria

#### 2.2.1. Diagnostic Criteria

Diagnostic criteria for oral diseases of “syndrome of fire-heat” by TCM were formulated as referred to by Diagnostics of TCM edited by Chen and Wilson [5].

1. Medical history: causative factors such as excessive spicy diet, tiredness, or excessive smoking and alcoholic drinking were found.
2. Symptoms: they are recurrent attacks of toothache, swelling and aching of gum, gingival bleeding, oral ulcers, halitosis, bitter taste of mouth and dry pharynx, and so forth, often accompanied by sore throat, hydrodipsia, constipation, and yellow urine.
3. Diagnosis: the patient has swelling and aching of gum, halitosis, red tongue, yellow coat, and red throat.

The above mentioned seven symptoms include toothache, swelling and aching of gum, gingival bleeding, aphthous stomatitis, halitosis, bitter taste of mouth, and dry pharynx; if the patients were with one of three (with three) or more they can be diagnosed as having “syndrome of fire-heat.”

#### 2.2.2. Inclusion Criteria

Inclusion Criteria encompass those who met the diagnostic criteria for oral diseases of “syndrome of fire-heat” by TCM; those who had no serious organic and mental diseases; those whose course of disease was within a month; those whose ages ranged from 18 to 55; those who had signed informed consent form (ICF). 

#### 2.2.3. Exclusion Criteria

The following subjects were excluded from this study: younger than 18 or older than 55; being pregnant or breast-feeding; being allergic to this toothpaste; used other brands of toothpastes for treatment; those who had complications from other diseases in cardiovascular system, cerebrovascular system, and hepatic, renal, and hematopoietic system as well as psychiatric patients.

### 2.3. Clinic Trial Design

We adopted a randomized, double-blind, single-center, and parallel control with positive toothpaste design for this study.

PROCPLAN procedure statement of SAS was used to design randomization of the trial. Statistical Analysis System (SAS) was software developed by North Carolina State University in 1966, with complete data access, data management, data analysis, and data presentation features. SAS was often used in the pharmaceutical industry for a variety of experimental studies, which could be done with simple programming. PROCPLAN procedure statement; of SAS was used to give a statement of randomized design. In this study, we gave a number of seeds randomly, and inputted statements meet the condition of this trail in the PROCPLAN procedure statement, then SAS outputted the therapeutic assignment corresponding to serial numbers from 01 to 72, which was, the randomized arrangement of treatments received by 72 subjects.

The requirement and design of blinding: verification toothpastes were packaged and provided by sponsor according to the randomization allocation table. The design of two-grade blind method was adopted, of which the first grade was the two treatments (group A or B randomly assigned) corresponding to each serial number from 01 to 72, while the second grade was the group (control group, treatment group) corresponding to A or B treatment. The allocation concealment of both grades was separately sealed up in duplicate for each, stored at the office of study site in the head organization and the sponsor. Two-grade unblinding was performed after cases collection. First of all, we clarified the code of two treatment groups corresponding to each serial number to perform statistical analysis and then identified the code of group (control group, treatment group) corresponding to each treatment group after completion of statistical analysis.

### 2.4. Treatment Methods

#### 2.4.1. The Name and Specification of Toothpaste Used in the Trial

Test toothpaste, Crest Herbal Crystal Toothpaste, as mentioned in Section 1, had the role of removing oral bacteria, freshening breath, and keeping health of teeth and gums, which was manufactured by P&G Technology Co., Ltd. (a research agency belonging to Procter & Gamble Company, which was mainly responsible for daily consumer goods' research and development, especially in the “fabric and home care” and “oral care” areas) batch no. 90991864BA, specification: 120 g/tube.

Control toothpaste: the selected Colgate Herbal Salty Toothpaste contains herbal essence to make oral cavity healthy and fresh of which the main ingredients were honeysuckle and mint. It has been in China market for a long time with the exact effect of “clearing internal heat,” which was manufactured by Colgate-Palmolive Co., Ltd. batch no. 9042CN11, specification: 120 g/tube.

#### 2.4.2. Package of Toothpaste

White toothpaste tube of qualified hygiene was adopted as package material for Crest Herbal Crystal Toothpaste and Colgate Herbal Salty Toothpaste, and the label are listed in Box 1.

#### 2.4.3. The Randomized Blinding

By using PROCPLAN procedure statement of SAS as mentioned in Section 2.3, we made randomly arrangements for treatments received by 72 subjects (test toothpaste or control toothpaste) that was to list the treatment allocation corresponding to serial numbers from 01 to 72.

#### 2.4.4. Allocation of Toothpaste

We distributed toothpaste corresponding to the code specified randomly which is screened to include the qualified subjects and to distribute toothpaste by the administrator according to the proper order of subject's visit as well as the sequence of toothpaste's code. “Registration table for product release” should be filled by administrator of toothpaste in time.

#### 2.4.5. Check Off Toothpaste

Whenever follow-up visit was made, the doctor should record the amount of toothpaste that the subject had received, used, and returned so as to determine the compliance of the subject in using the toothpaste.

#### 2.4.6. Preservation of Toothpaste

The toothpastes used in the trial were preserved by designated person at dry drafty place under room temperature and distributed to the subjects according to the requirement of the trial design.

#### 2.4.7. Toothpastes Used in Combination

1. Except the toothpastes used for verification, other therapies related to the treatment of the diseases were not allowed to use during the observation period.
2. If medicines or other therapies must be used for complicated diseases, then the drug names (or names of other therapies), dosage, frequency, and time of usage must be noted on the medical record for the trial in order to be used for analysis and report at summarization.

#### 2.4.8. Treatment and Observation Time for Subjects

Inclusion of subject: subjects who met the inclusion criteria, being out of exclusion criteria, were included for observation.

Treatment methods: Crest Herbal Crystal Toothpaste was provided for test group, while Colgate Herbal Salty Toothpaste was provided for control group, and both groups were given 1 g/each time, bid. The subjects were instructed in correct brushing method (BASS brushing method, brushed teeth twice a day, morning and night, at least 2 minutes for tooth-brushing each time). (The subjects were required to be present at trial site receiving tooth-brushing instruction for 3 times every week.) All subjects spent 4 weeks (28 d) for treatment course.

The Bass brushing technique is scientifically proven to disrupt, disorganize, and remove the bad bugs that cause gum disease in our mouth. The Bass method, or Sulcular vibration brushing, or the 45-degree angle tooth brushing technique is a very effective method for germs or plaque removal next to and directly below the gum or gingival margin. The area at the gum-tooth margin is the most significant in the prevention of tooth decay and gum disease [6].

Observation time: the rating scale (the 3rd edition) [4] on efficacy of “clearing internal heat” for the herbal toothpaste was used before toothpaste application, at 2 w (14 d) of application and 4 w (28 d), respectively, for the subjects to fill for 3 times separately.

### 2.5. Criteria of Efficacy

#### 2.5.1. Measurement

Rating scale (the 3rd edition) [4] on efficacy of “clearing internal heat” for the herbal toothpaste is one of the criteria.

#### 2.5.2. Criteria for General Efficacy Determination

Refer to Guiding Principles for the New Drug Clinical Research of China Medicines (On trial) [7]. Symptomatic quantifying standards: 4-level method is adopted for grading: 0 point for absence of symptoms; 2 points for slight level; 4 points for mediate level; 6 points for severe level.

*N* represents curative index: *N* = ((accumulated points of symptoms before treatment − accumulated points of symptoms after treatment)/accumulated points of symptoms before treatment) × 100%.

Clinical cure: symptoms and signs disappeared after treatment, *N* ≥ 95%; marked improvement: most of symptoms and signs disappeared after treatment, 95% > *N* ≥ 70%; effective: a part of symptoms and signs disappeared after treatment, 70% > *N* ≥ 30%; ineffective: symptoms and signs did not disappear or aggravated after treatment, *N* < 30%.

#### 2.5.3. Criteria of Efficacy Determination for Single Parameter

*N* represents curative index: *N* = ((grade of symptoms before treatment − grade of symptoms after treatment)/grade of symptoms before treatment) × 100%.

Cure: *N* = 1; marked improvement: 1 > *N* = 2/3 (67%); effective: *N* = 1/3 (33%) or *N* = 1/2 (50%); ineffective: *N* = 0 or *N* < 0.

### 2.6. Statistical Analysis

#### 2.6.1. Choice of Statistical Data for Evaluation of Curative Effect

Intention-to-treat (ITT) analysis and per-protocol (PP) analysis was adopted. ITT analysis could prevent the poor prognosis patients excluded from the analysis and retain the advantages of randomization; PP analysis could reflect the actual results of completion of treatment by program and reduce the impact of interference or contamination.

Full analysis set: all randomized subjects were included, and those subjects without any observation data for follow-visits were rejected. For the data of subjects which did not include all results of entire treatment course, the result for latest observation must be carried forward to where the absence of verification data was found. The number of subjects at the endpoint of curative evaluation should be the same as that at the start of trial. PP set includes subjects who were consistent with the trial protocol, while the major variables were measurable, and no great violation to trial protocol was found.

ITT analysis was performed on 72 subjects, who were on full analysis set, of which 24 subjects were from group A and 48 subjects were from group B; 67 subjects were on PP set, of which 24 subjects were from group A and 43 subjects were from group B; 5 subjects dropped out due to illness or official business and private business.

In this study, the results would be better reflected when we performed the ITT and PP analysis at the same time. The closer the ITT and PP results are, the less the proportion of defaulter, the higher the quality of research, and the more credible the results will be.

#### 2.6.2. Statistical Method

As for descriptive statistical analysis, qualitative indexes were described with percentage or constituent ratio, while quantitative indexes were described with mean ± standard deviation (SD). In comparative analysis between the two groups, we used Chi-square test, Fisher's exact test, and Wilcoxon rank sum tests. *t* test was performed on quantitative data of normal distribution, and Wilcoxon rank sum test was done on data with skewed distribution data.

For demographic data analysis, Chi-square analysis was conducted to compare the sex ratio of two groups, and *t* test was done to compare age and baseline inner heat level. For efficacy data analysis, normal test was done firstly to check if data fit in normal distribution, and then *t* test or Wilcoxon rank sum test was done based on data property. Unified double-tailed test was used for hypothesis testing, while test statistics and the corresponding *P* value were provided, and the statistical significance was confirmed if *P* < 0.05. EXCEL, Epidata 3.02, or SAS 8.2 statistical package was used in above analysis.

## 3. Results

**Figure 1:**
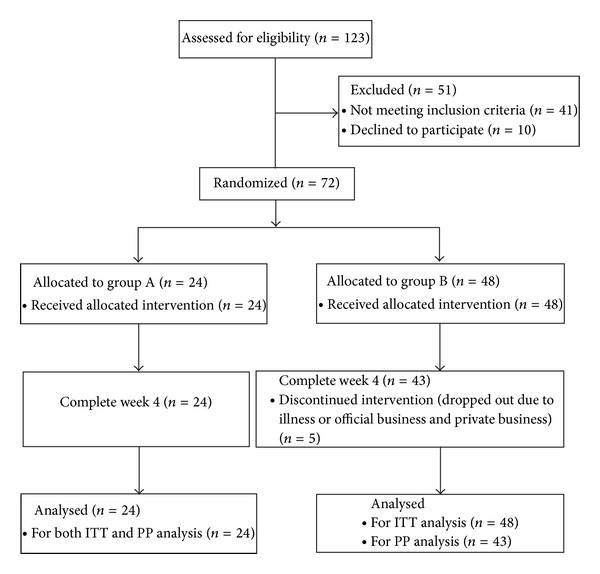
Participant flow through recruitment to trial completion.

A total of 123 subjects were assessed for eligibility, and 72 subjects were enrolled, 24 were randomised to group A and 48 to the control group B. Five randomly allocated participants in group B did not complete the study (see Figure 1).

### 3.1. Comparison of Demographic Data

Baseline characteristics are listed in Table 1. There was no significant difference between the two groups on sex, age, and level of fire-heat syndrome.

In Table 1, the difference of the demographic data such as sex, age and level of fire-heat syndrome among subjects between the two groups was not statistically significant (*P* > 0.05), which indicated the same baseline of two groups, and available comparability.

### 3.2. Evaluation Result from the Rating Scale (the 3rd Edition) [4] on Efficacy of “Clearing Internal Heat” for the Herbal Toothpaste

#### 3.2.1. Evaluation Result of Full Analysis Set for 72 Subjects in the Two Groups (Intention-to-Treat (ITT) Analysis)

It was shown in Table 2 that the mean value of syndrome of fire-heat has been reduced for both groups A and B, and there was no difference in curative effect between the two groups. Equivalence test was performed on the two groups at the same time, and the result 17.73 was between *Q* = 13.84~25.24, indicating that the two groups were equivalent in treatment effect.

#### 3.2.2. Per-Protocol (PP) Analysis for 67 Subjects of the Two Groups

It was shown in Table 3 that the mean value of syndrome of fire-heat has been reduced for both groups A and B, and there was no difference in curative effect between the two groups. The result 17.40 was between *Q* = 13.76~25.32, indicating that the two groups were equivalent in treatment effect.

The results in Tables 2 and 3 indicated that from the rating scale (the 3rd edition) [4] on efficacy of “clearing internal heat” for the herbal toothpaste, the original accumulated points were found to be reduced progressively with statistical significance for subjects of both group A and group B after application of toothpaste, which suggested that symptoms of fire-heat have been improved for both groups after use of the toothpaste. The results were consistent with ITT and PP analysis.

#### 3.2.3. Evaluating the Curative Effect for the Two Groups after Treatment with the Rating Scale (the 3rd Edition) [4]

With effective rate ≥ 33%, it meant effective under this item; otherwise it was ineffective. It was shown in Table 4 that in group A, the effective rate of *Q*5, *Q*7, and *Q*8 was greater than 33%. In group B, the effective rate of *Q*2~*Q*8 was greater than 33%. The overall effective rate of both groups was slightly lower than 33%, and no significant difference was found in curative effect between the two groups (*P* > 0.05).

It was shown in Table 5 that in group A, the effective rate of *Q*5, *Q*7, and *Q*8 was greater than 33%, which represented effectiveness, while the rest was lower than 33%, which represented ineffectiveness, and the overall effective rate was 31% < 33%. In group B, the effective rate of *Q*2~*Q*8 was greater than 33%, which represented effectiveness, while the rest was lower than 33%, which represented ineffectiveness, and the overall effective rate was 34% > 33%. No significant difference was found in curative effect between the two groups (*P* > 0.05).

From the rating scale (the 3rd edition) [4] on efficacy of “clearing internal heat” for the herbal toothpaste, different data analysis was used in Tables 4 and 5, and the result indicated that the overall effective rate for both group A and group B was about 33%, which represented effectiveness.

#### 3.2.4. Comparison of Curative Effect of the Two Groups with the Rating Scale (the 3rd Edition) [4] after the Treatment

Table 6 showed that effective rate in group A was slightly higher than that in group B (both values ≥30%), while the ineffective rate in group A was slightly lower than that in group B, and *P* > 0.05 by Wilcoxon rank sum test for both groups. No statistical significance was found, indicating there was no difference of overall curative effect between the two groups. The results were consistent with ITT and PP analysis.

## 4. Discussion

Oral diseases such as toothache, sore, or bleeding gums are very common, and most of them are regarded as “excessive internal heat” by TCM. The symptom of fire-heat is classified as deficiency and excess, where the fire in ZANG FU-organ is divided into stomach-fire, excessive heart fire, and hepatic fire. The formation of oral fire-heat symptom is related to the factors such as excessive spicy diet, tiredness, or excessive smoking and alcoholic drinking, as well as long-term depressed emotion leading to fire symptom [5]. However, in consideration of the manifestations of oral fire-heat symptom especially in young subjects, it mainly belongs to stomach-fire pattern manifestation such as toothache, swelling and aching of gum, gingival bleeding, oral ulcers, halitosis, bitter taste of mouth, and dry pharynx often accompanied by sore throat, hydrodipsia, constipation and yellow urine, and red tongue with thick yellow coat [5].

Based on the TCM theory, patients who suffered from oral fire-heat pattern could be easily diagnosed by a TCM doctor according to medical history and symptoms. However, such oral fire-heat pattern was often neglected by the patients, so we have developed a self-report rating scale (the 3rd edition) [4] (with good reliability and validity) to objectively evaluate oral fire-heat pattern. Crest Herbal Crystal Toothpaste, containing herbs of honeysuckle and mint with heat-clearing and detoxing, functions can protect teeth and gums. Honeysuckle, Flos Lonicerae, is a medically useful TCM herb, with the function of clearing heat and removing toxicity and of antibacterial activity [8]. Mint, *mentha haplocalyx*, is also commonly used in TCM, with the function of eliminating pathogen, in addition to fresh breath [8]. Also Colgate Herbal Salty Toothpaste with the efficacy of “clearing internal heat” is designated as positive control in the trial. Both herbal toothpastes with exotic herbal ingredients for tingly fresh breath and all day protection contain nature's best herbs which soothe oral ailments like toothaches, mouth ulcers, and inflamed gums. 

Evaluation with the rating scale (the 3rd edition) [4] on efficacy of “clearing internal heat” for the herbal toothpaste indicated that no significant difference between Crest Herbal Crystal Toothpaste and the positive control was found in aspect of improving the accumulated points of oral fire-heat syndrome, for the subjects, level of oral fire-heat syndrome and overall effective rate. Meanwhile, we simultaneously observed the evaluation by the doctor with the symptoms grading scale of fire-heat pattern (SGS-FHP) for the herbal toothpaste, and the results also indicated that no significant difference between Crest Herbal Crystal Toothpaste and the positive control was found (this will be published in another report). This suggested that Crest Herbal Crystal Toothpaste has the equal efficacy of “clearing internal heat”, while the availability of efficacy of “clearing internal heat” for Crest Herbal Crystal Toothpaste can be evaluated objectively by the rating scale (the 3rd edition) [4] on efficacy of “clearing internal heat” for the herbal toothpaste.

## 5. Conclusion

This study confirms that the Crest Herbal Crystal Toothpaste has the effect of “clearing internal heat” with Colgate Herbal Salty Toothpaste as the on-market control. And the efficacy of Crest Toothpaste in “clearing internal heat” is evaluated by the rating scale (the 3rd edition) [4] on efficacy of “clearing internal heat” for the herbal toothpaste.

## Figures and Tables

**Box 1:**
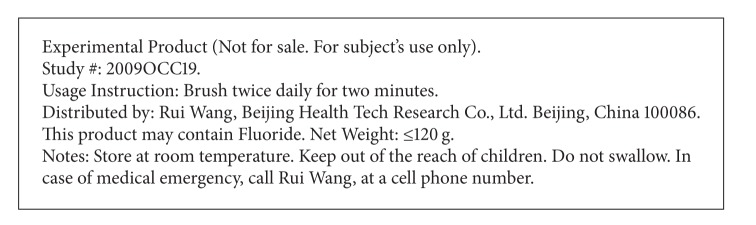
Description for package of toothpaste used in the trial.

**Table 1:** Comparison of demographic data between the two groups (mean value ± standard deviation).

Group	Sex (male/female)	Age (*X* ± SD)	Grade of symptoms (*X* ± SD)
Group A (24 subjects)	7/17	28.42 ± 7.52	28.33 ± 6.79
Group B (48 subjects)	9/39	27.88 ± 6.99	26.25 ± 4.88
*P* value	0.3162 (*X* ^2^ = 1.0045)	0.6789 (*Z* = 0.4140)	0.2337 (*Z* = 1.1908)

**Table 2:** Comparison of original accumulated points among 72 subjects between the two groups before and after treatment (mean value ± standard deviation) (intention-to-treat (ITT) analysis).

Group	Baseline	Week 2	Week 4
Group A (24 subjects)	28.33 ± 6.79	21.75±6.04*	19.54±6.19*
Group B (48 subjects)	26.25 ± 4.88	20.02±5.72*	17.73±5.48*

Compare with the result before treatment in the group, *P < 0.05.

**Table 3:** Comparison of original accumulated points among 67 subjects between the two groups before and after treatment (mean value ± standard deviation) (per-protocol (PP) analysis).

Group	Baseline	Week 2	Week 4
Group A (24 subjects)	28.33 ± 6.79	21.75±6.04*	19.54±6.19*
Group B (43 subjects)	26.34 ± 4.82	19.95±5.50*	17.40±5.22*

Compare with the result before treatment in the group, *P < 0.05.

**Table 4:** Effective rate of full analysis set for 72 subjects in the two groups after treatment (%, ITT).

	Q1	Q2	*Q*3	*Q*4	*Q*5	*Q*6	*Q*7	*Q*8	*Q*9	*Q*10	*Q*11	(total) *Q *
Group A (24 subjects)	26%	31%	26%	30%	41%	32%	40%	37%	19%	30%	23%	31%
Group B (48 subjects)	29%	36%	41%	33%	39%	35%	33%	38%	23%	12%	22%	32%

Mean = − 0.0058; SD *⁡* = 0.078; *t* = −0.2597; *P* > 0.05.

**Table 5:** Effective rate of PP set for 67 subjects in the two groups after treatment (%, PP).

	*Q*1	*Q*2	*Q*3	*Q*4	*Q*5	*Q*6	*Q*7	*Q*8	*Q*9	*Q*10	*Q*11	(total) *Q *
Group A (24 subjects)	26%	31%	26%	30%	41%	32%	40%	37%	19%	30%	23%	31%
Group B (43 subjects)	31%	38%	43%	35%	43%	36%	33%	39%	24%	15%	22%	34%

Mean = − 0.0225; SD = 0.077; *t* = −1.006; *P* > 0.05.

**Table 6:** Evaluating the overall effective rate of both groups with the rating scale (the 3rd edition) [4] after treatment (number of cases and %).

Dataset	Group	Cure	Markedly effective	Effective	Ineffective	
FA set	Group A (24 subjects)	0 (0)	0 (0)	15 (62.50)	9 (37.50)	*z* = −0.4613
(ITT)	Group B (48 subjects)	0 (0)	0 (0)	27 (56.25)	21 (43.75)	*P* = 0.6446
PP set	Group A (24 subjects)	0 (0)	0 (0)	15 (62.50)	9 (37.50)	*z* = −0.4613
(PP)	Group B (43 subjects)	0 (0)	0 (0)	26 (60.64)	17 (39.53)	*P* = 0.6446

(Full analysis) FA set *P* = 0.6446,  PP set *P* = 0.6446.

## Appendix

### The Rating Scale (the 3rd Edition) [4] on Efficacy of “Clearing Internal Heat” for the Herbal Toothpaste

Self-filling section: the following questions inquire about the common symptoms of traditional Chinese medicine during the last month. Answer every question by marking the appropriate box with a “*√*”. Please according to your true feelings choose from one of the following answers.

**Table:**

	1	2	3	4	5
	Never	Occasionally	Often	Very often	Always
How often was it, that you (your)	1	2	3	4	5
(1) feel dryness of throat;	□	□	□	□	□
(2) feel thirsty and want to drink especially cold water;	□	□	□	□	□
(3) feel bitter taste in mouth;	□	□	□	□	□
(4) suffered from oral malodor (halitosis);	□	□	□	□	□
(5) suffered from oral ulcer on mucosa or tongue;	□	□	□	□	□
(6) suffered from swelling and aching of gums;	□	□	□	□	□
(7) suffered from bleeding gums;	□	□	□	□	□
(8) suffered from toothache;	□	□	□	□	□
(9) suffered from lightly swelling, but no red or aching of gums;	□	□	□	□	□
(10) suffered from loose teeth and inability to chew;	□	□	□	□	□
(11) suffered from dry teeth (sticky feeling between the teeth and upper and lower lips).	□	□	□	□	□

Interview section: tongue body and tongue coating inspected and chose by TCM doctors.

(12) Tongue body: pale, pink, red, light purplish, deep red, or fissured.
(13)  Tongue coating: thin and whitish, white, thin and yellowish, yellowish greasy, dryness, eroded, or less coating.
